# Protective immunity to Japanese encephalitis virus associated with anti-NS1 antibodies in a mouse model

**DOI:** 10.1186/1743-422X-9-135

**Published:** 2012-07-24

**Authors:** Yize Li, Dorian Counor, Peng Lu, Veasna Duong, Yongxin Yu, Vincent Deubel

**Affiliations:** 1 Key Laboratory of Molecular Virology and Immunology, Institut Pasteur of Shanghai, Shanghai Institutes for Biological Sciences, Chinese Academy of Sciences, Shanghai, 20025, China; 2 Unit of Virology, Institut Pasteur in Cambodia, Phnom Penh, Cambodia; 3 National Institute for the Control of Pharmaceutical and Biological Products, Tiantan Xili Chongwen Qu, Beijing, 100050, China; 4 Present address: Institut Pasteur in Cambodia, Phnom Penh, Cambodia

**Keywords:** Japanese encephalitis virus, T-cell response, Antibodies, Monoclonal antibodies, NS1 protein, Mouse model

## Abstract

**Background:**

Japanese encephalitis virus (JEV) is a major mosquito-borne pathogen that causes viral encephalitis throughout Asia. Vaccination with an inactive JEV particle or attenuated virus is an efficient preventative measure for controlling infection. Flavivirus NS1 protein is a glycoprotein secreted during viral replication that plays multiple roles in the viral life cycle and pathogenesis. Utilizing JEV NS1 as an antigen in viral vectors induces a limited protective immune response against infection. Previous studies using *E. coli*-expressed JEV NS1 to immunize mice induced protection against lethal challenge; however, the protection mechanism through cellular and humoral immune responses was not described.

**Results:**

JEV NS1 was expressed in and purified from *Drosophila* S2 cells in a native glycosylated multimeric form, which induced T-cell and antibody responses in immunized C3H/HeN mice. Mice vaccinated with 1 μg NS1 with or without water-in-oil adjuvant were partially protected against viral challenge and higher protection was observed in mice with higher antibody titers. IgG1 was preferentially elicited by an adjuvanted NS1 protein, whereas a larger load of IFN-γ was produced in splenocytes from mice immunized with aqueous NS1. Mice that passively received anti-NS1 mouse polyclonal immune sera were protected, and this phenomenon was dose-dependent, whereas protection was low or delayed after the passive transfer of anti-NS1 MAbs.

**Conclusion:**

The purified NS1 subunit induced protective immunity in relation with anti-NS1 IgG1 antibodies. NS1 protein efficiently stimulated Th1-cell proliferation and IFN-γ production. Protection against lethal challenge was elicited by passive transfer of anti-NS1 antisera, suggesting that anti-NS1 antibodies play a substantial role in anti-viral immunity

## Introduction

Japanese encephalitis virus (JEV) is one of the most important mosquito-borne viruses in East and Southeast Asia, where seasonal outbreaks cause more than 50,000 infections and 10,000 deaths annually [1]. JEV is a mosquito-borne flavivirus in the family Flaviviridae. The *Flavivirus* genus contains more than 70 viruses with positive-sense, single-stranded RNA genomes (~11 kb) that encode a polypeptide (~ 3400 amino acids) consisting of the capsid protein C (core protein), the matrix protein (envelope protein M), the major envelope protein E, a number of small non-structural proteins (NS1, NS2A, NS2B, NS4A and NS4B), a helicase (NS3) and a RNA-directed polymerase (NS5) that are cleaved and co- or post-translationally processed by host- or virus-specific proteases [2]. The first non-structural protein (NS1) is translocated to the endoplasmic reticulum (ER) via signal sequences in a trans-membrane C-terminal stretch of protein E, where it is involved in ER-associated RNA replication [3]. NS1 is N-glycosylated then secreted to the extracellular milieu [4]. The pathogenic role of NS1 remains largely unknown, but has been shown to be associated with Factor H in West Nile virus (WNV) infection [5] and with C4b in WNV, yellow fever virus (YFV), and dengue virus (DENV) infections, where it may modulate complement activation [6,7]. NS1 may use structural mimicry similar to those found in the endothelial membrane or coagulation factors, which may elicit the autoimmunity that is deleterious in DENV hemorrhagic fever [8,9]. However, no such auto-antibody has yet been found in JEV infection [10]. Its association with phospholipids may induce vascular homeostasis in DENV hemorrhagic fever, which is similar to that induced by plasma lipoproteins [4].

Prevention of Japanese encephalitis through vaccination was shown to be efficient when using either a formalin-inactivated virus produced in mouse brain or cell culture, or a live attenuated vaccine, SA14-14-2 [11], which was developed in China and is broadly used for childhood vaccination in mainland China, India, and several other Southeast Asian countries [12]. The SA14-14-2 virus is produced in hamster primary kidney cells and is widely used in vaccination programs because of its innocuity, efficiency, and low cost to developing countries. A JEV vaccine approved by the Food and Drug Administration was recently produced in Vero cells, which was purified from SA14-14-2-infected cell supernatants and inactivated formalin [11].

The immunogenicity and protective immunity of flavivirus NS1 has been studied using various vaccine types. Animals were either immunized with YFV NS1 [13], or infected with vaccinia viruses or adenoviruses expressing recombinant NS1 from DENV [14], YFV [15], tick-borne encephalitis virus (TBEV) [16,17], or WNV [18]. However, they showed variable protective immune responses. Vaccinations with flavivirus NS1 DNA were also tested in NS1-induced immune protection studies against DENV [19] and TBEV [20]. These studies showed that the NS1 contributes to the induction of protective immunity against flaviviral infections. The anti-NS1 protection mechanism was partially determined, showing that passive transfer of anti-YFV NS1 monoclonal antibodies (MAbs) protected mice or reduced their neuropathology after YFV challenge [21,22] and that WNV anti-NS1 MAbs protected mice from lethal challenge [23,24].

Mice immunized with JEV NS1 expressed in insect cells induced low protection [25]. Recombinant viruses or DNA vaccines expressing JEV E or NS1 genes were used to vaccinate mice, which resulted in differing levels of protection [26]. The value of using a vaccine containing the native hexameric form of a purified flavivirus NS1 glycoprotein to protect against lethal viral challenges has yet to be tested in mice. One obstacle in protection studies is that mice are less susceptible to a viral challenge by the time a vaccination schedule in immunocompetent mice is achieved. A possible surrogate for testing protective efficacy is the use of adoptive immunity by injecting flavivirus anti-NS1 antibodies into young mice [14,17,22].

A JEV NS1 protein purified from a *Drosophila* S2 established cell line supernatant [27] was used to immunize C3H/HeN (C3H) mice and to test the induction of cellular and humoral immunity. Protection of mice by antibodies elicited against NS1 was tested by JEV infection of mice that were actively or passively immunized. Our results support the role of anti-NS1 antibodies in protection against flaviviral infection.

## Results

### Characterization of the NS1 immunogen

In a previous study, we successfully produced and purified large amounts of extracellular hexameric forms of JEV NS1 protein [27]. We studied the processing and composition of carbohydrates of NS1 protein produced in the supernatant of *Drosophila S2* cells. Heat-denatured NS1 was mock-treated or treated with Endo H (Endoglycosidase H) or PNGase F (Peptide: N-Glycosidase F). Endo H and PNGase F treatment reduced the molecular weight of extracellular NS1, by 2 kD (Additional file 1: Figure S1A), indicating that the protein acquired glycans in the secretory pathway of *Drosophila* cells. The type of glycans was identified by lectin affinity. The NS1 protein was recognized by GNA (*Galanthus nivalis* agglutinin) which recognizes terminal mannose, and DSA (*Datura stramonium* agglutinin) which recognizes Gal-(1–4)N-Acetylglucosamine (GlcNAc) in complex and hybrid N-glycans, respectively (Additional file 1: Figure S1B), but not by SNA (*Sambucus nigra* agglutinin) which recognizes sialic acid linked (2–6) to galactose and MAA (*Maackia amurensis* agglutinin) which recognizes sialic acid linked (2–3) to galactose (data not shown). These results indicated that the glycoprotein NS1 contained N-glycans with Gal-(1–4)GlcNAc and a terminal mannose, but apparently not sialic acid.

### Mouse immune responses to NS1 and SA14-14-2 immunization

**Figure 1:**
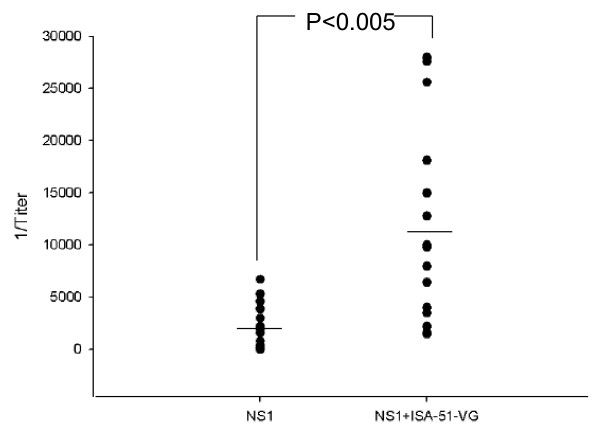
**IgG antibody response of mice immunized with aqueous and adjuvanted NS1.** Four-week-old C3H mice were immunized with 1 μg of purified NS1. Two weeks after a second immunization was given at two months of age, mouse sera were sampled and the IgG titers were tested by indirect ELISA. The data for immunization with recombinant NS1 are derived from two independent experiments. Antibodies of 18 mice sera were titrated for each group and analyzed by Student’s *t-*test.

**Figure 2:**
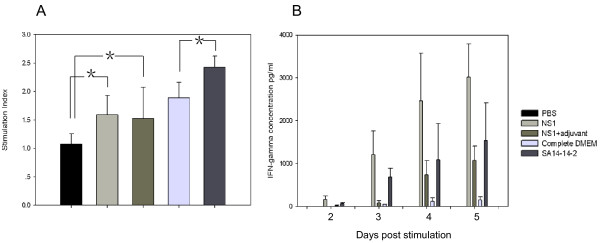
**Proliferation and IFN-γ secretion of splenic cells from immunized C3H mice in response to inoculation with recombinant JEV NS1 protein and SA14-14-2.** Splenocytes isolated from mice immunized with PBS, aqueous NS1, adjuvanted NS1, DMEM with 3% FBS (complemented DMEM) and attenuated virus SA14-14-2, were stimulated with purified NS1. **A:** Splenocyte proliferation. The proliferation stimulation index (SI) was defined as the ratio of stimulated cells to unstimulated cells. **B:** IFN-γ secretion. The IFN-γ production of splenic cells was quantified over a period of five days. Three mice were used for each group. The SI and IFN-γ concentration were analyzed by Student’s *t-test*. *P* < 0.05 was considered to be significant. *, significant difference.

Four-week-old C3H mice were immunized twice four weeks apart using a purified NS1 hexamer obtained from *Drosophila* S2 cell line supernatants [27], either alone (aqueous) or adjuvanted with water-in-oil ISA-51-VG. Serum samples from each group were tested individually by anti-NS1 antibody titration. NS1 immunization induced anti-NS1 antibody responses in both groups of mice and the use of an adjuvant significantly increased the antibody titer (*p* < 0.005) (Figure 1). Six mouse sera samples from each group were pooled to test IgG1 and IgG2a antibodies against NS1. Immunization with aqueous or adjuvanted NS1 protein induced both IgG1 and IgG2a antibody responses, with an IgG1 titer four to nine times higher than that of IgG2a, but a similar titer to IgG2a (Table 1). SA14-14-2 vaccination mainly induced anti-NS1 IgG2a antibodies. The proliferation of T-cells and IFN-γ secretion by splenic cells of immunized C3H mice in response to stimulation with purified JEV NS1 protein were tested. The T-cell proliferation stimulation index (SI) of the PBS group was 1, while those of the aqueous NS1 and adjuvanted NS1 groups were 1.59 and 1.51, respectively, and these were both significantly higher (*p* < 0.05) than the SI of the phosphate buffered saline (PBS) group (Figure 2A). After vaccination with SA14-14-2 or Dulbecco's modified Eagle's medium (DMEM), purified NS1 was used for splenic cell stimulation in vitro. The T-cell proliferation SI of SA14-14-2 was 2.42, significantly higher than the control group (SI = 1.88; *p* < 0.05) (Figure 2A). IFN-γ production by splenic cells was quantified over a period of five days. IFN-γ was detected on day 2 or 3 post-stimulation in the splenocyte culture supernatants of mice immunized with aqueous or adjuvanted NS1, SA14-14-2 and complete DMEM. The highest IFN-γ load was 3 ng ml^–1^ in the aqueous NS1 group, 1 ng ml^–1^ in the adjuvanted NS1 group, 1.5 ng ml^–1^ in the SA14-14-2 group and 150 pg ml^–1^ in the complete DMEM group on day 5 post-stimulation (Figure 2B).

**Table 1:** Determination of antibodies titers and sero-neutralization of pooled sera from immunized mice

**Immunogen**	**Antibodies Titration**						**Sero- IgG IgG1 IgG2a neutralization**	
	**IgG**		**IgG1**		**IgG2a**			
	Before^a^	After^b^	Before	After	Before	After	Before	After
NS1	5000	6400	5000	5000	1200	3000	0	40
NS1 + adjuvant	15000	18000	15000	15000	1600	3200	0	40
SA14-14-2	1200	1000	300	0	2400	2400	80	80
PBS	0	600	0	0	0	2400	0	40

^a^ Sera collected two weeks after 2^nd^ boost and two weeks before challenge.

^b^ Sera collected four weeks after challenge.

### NS1 immunization induced immune responses that partially protected mice from viral challenge

**Figure 3:**
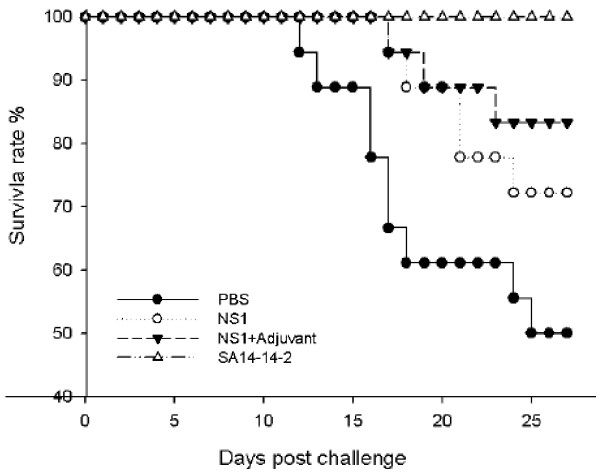
**Survival curves of SA14-14-2-vaccinated and NS1-immunized mice after JEV challenge.** Groups of three-month-old SA14-14-2-vaccinated or NS1-immunized C3H mice (see Figure 1) were i.n. challenged with 1 × 10^4^ pfu of JEV SA14. The JEV-infected mice were monitored daily for 28 days. Survival curves of mice immunized with recombinant NS1 protein or injected with PBS were constructed using data from two experiments, with groups of 6–12 mice. JEV-vaccinated mice were tested once using eight mice. Control mice received PBS and were challenged with JEV.

To determine whether the anti-NS1 immunity observed in immunized mice was protective, mice were challenged by an intranasal (i.n.) injection of 10^4^ pfu of JEV SA14 and mortality was recorded over four weeks . This virus dose corresponded to 100 times the lowest dose killing the largest number of mice (Additional file 2: Figure S2B). Mouse challenge by i.n. was chosen rather than i.p. because the number of deaths and the time before death were more consistent (Additional file 2: Figure S2A). The survival rate of the adjuvanted NS1 group was significantly higher (83%) when compared with a negative control group (injection with PBS) that had a 50% survival rate (*p* < 0.05) (Figure 3, Table 2). The survival rate of the aqueous NS1 group was 72%, but the difference from the survival rate of the negative control group was not significant (*p* > 0.05) (Table 2), although a delay in the time of death was observed in the aqueous NS1-immunized mice when compared with the PBS mock-immunized mice (Figure 3).

**Table 2:** Survival rate of C3H mice immunized with NS1 and SA14-14-2

**Immunogen**	**No. of Survival mice/ No. of Immunized mice (survival rate)**	***p***^**a**^
NS1	13/18 (72%)	0.195
NS1 + adjuvant	15/18 (83%)	0.024
SA14-14-2	8/8 (100%)	0.021
PBS	9/18 (50%)	

^a^*P* value was obtained by log rank test when comparing the survival curve of each group of vaccinated mice with PBS group.

Sera of mice that had survived the SA14 virus challenge were titrated by ELISA. SA14 infection did not increase the anti-NS1 IgG2a titers in the SA14-14-2-vaccinated group, and anti-NS1 IgG1 was not detected after the challenge. In contrast, SA14 challenge induced an IgG2a antibody titer increase, but not IgG1, in the aqueous or adjuvanted NS1 groups (Table 1). Mouse sera collected before and after challenge were also tested for seroneutralization to follow seroconversion. Sera from mice immunized with NS1 reached a TCID_50_ titer of 40 after the challenge. Sera from the SA14-14-2 group exhibited the same neutralization titer (TCID_50_ titer = 80) before and after the challenge (Table 1). These data suggest that the attenuated virus vaccine, but not NS1, induced a sterilizing immunity against JEV.

In a previous experiment, sera from mice were pooled for antibody subtyping and neutralization testing. In order to verify whether there was any correlation between anti-NS1 antibody titer elicited by vaccination with NS1 and challenge protection, ELISA titers of 18 mice were calculated individually before challenge. Table 3 shows that the total number of surviving aqueous NS1-vaccinated mice (66.7%) was not significantly higher than those receiving PBS (44.4%) (p = 0.41); whereas all mice that had anti-NS1 antibody titers ≥ 2400 were all protected (p = 0.008) compared to mice that had titers < 2400 were not protected (33.3%) compared to the control (44.4%) (*p* = 1). This result suggests a relationship between anti-NS1 antibody titer and protective immunity against i.n. JEV challenge.

**Table 3:** Survival rate of three month-old C3H mice immunized with NS1 or PBS

**Immunogen**	**Anti-NS1 IgG antibody titer range**^**a**^	**No. of Survival mice/No. of Immunized mice (Survival rate)**	***P***^**b**^
NS1	0-12800	12/18 (66.7%)	0.41
NS1	0-2400	3/9 (33.3%)	1
NS1	2400-12800	9/9 (100%)	0.008
PBS	0	4/9 (44.4%)	

^a^ Two weeks before challenge, mouse sera were collected for anti-NS1 IgG antibody titration. The NS1-immunized group of mice (18 mice in total) was divided into two groups according to anti-NS1 IgG titers: one group with anti-NS1 IgG <2400 (9 mice) and one group with anti-NS1 IgG >2400 (9 mice).

^b^*P* value was obtained by log rank test when comparing the survival curve of each group with PBS group.

### Passive protection of mice by anti-NS1 antibodies

We previously calculated that 20 pfu of JEV SA14 virus could kill 100% of four-week-old i.p.-infected C3H mice [28] as shown in Additional file 2: figure S2C.

**Figure 4:**
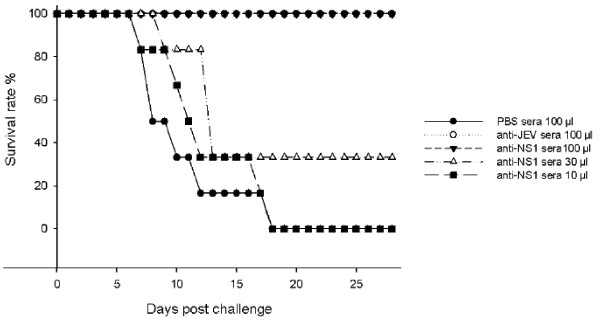
**Survival of mice passively injected with anti-NS1 antisera and challenged with JEV.** Groups of four-week-old C3H mice were administered with 100 μl, 30 μl and 10 μl of anti-NS1 mouse antisera, or 100 μl sera from PBS-injected mice (negative control) or 100 μl of anti-SA14-14-2 antisera (anti-JEV), and infected 1 hr later with 20 pfu of JEV SA14. The JEV infected mice were monitored daily for 28 days. The survival curve was constructed using data from one (100 μl anti-JEV, 30 and 10 μl of anti-NS1 antisera), two (sera from PBS-injected mice) or three (100 μl of anti-NS1 antisera) experiments. For 100 μl anti-JEV antisera group, 30 and 10 μl of anti-NS1 antisera groups, sera from PBS-injected mice group, 6 mice were used for each experiments. Six to 12 mice were used for 100 μl anti-NS1 antisera groups of each experiment.

To determine whether the protection of immunized mice was associated with an anti-NS1 antibody response, the sera from NS1-immunized mice with high titers of anti-NS1 antibodies (>1:3000) were collected and pooled to perform passive transfer tests. One hundred μl of NS1 mouse anti-sera injected 1 h prior to challenge provided 100% protection in challenged mice, but decreasing the dose to 30 μl and 10 μl provided 33% and no protection, respectively (Figure 4). While sera from SA14-14-2-vaccinated mice conferred 100% protection, all mice died that were injected with sera from PBS mock-immunized mice (Figure 4).

### Characterization of a panel of MAbs against NS1, for passive protection

**Figure 5:**
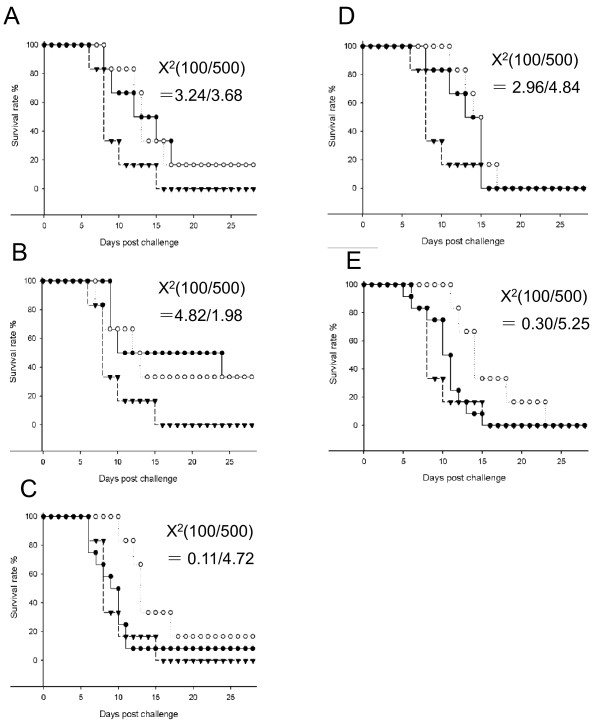
**Survival of mice passively immunized with anti-NS1 monoclonal antibodies.** Groups of four-week-old C3H mice were administered with 100 μg (black dot) or 500 μg (white dot) of purified MAbs 3E10 **(A)**, 4C4 **(B)**, 7C2 **(C)**, 7H5 **(D)**, 8F1 **(E)** before being challenged with 20 pfu of SA14. Another group of mice received 100 μg of HIV anti-envelope MAb (black triangle) as a negative control. JEV-infected mice were monitored daily for 28 days post-challenge. Survival curves were constructed using data from one or two experiments with 6–12 mice in each group. χ^2^ values were obtained using a log-rank test by comparing the survival curve of each group with the anti-HIV group.

Eighteen MAbs against NS1 were generated in a previous study [27] and five had high affinity for NS1 (3E10, 4C4, 7C2, 7H5 and 8F1), where they recognized cell surface-associated and intracellular NS1 (Table 4). MAb 4C4 recognized the N-terminus of NS1; MAb 3E10 detected NS1 in an ELISA and IFA, but not in a Western blot, indicating a probable recognition of a conformational epitope. Three MAbs recognized the C-terminus of the protein and cross-reacted with the DENV NS1 protein (Table 4). These five MAbs were purified and quantified before being passively transferred at doses of 100 μg or 500 μg to four-week-old mice. Mice were challenged with 20 pfu of SA14 virus 1 h after MAb injection and mortality was recorded for four weeks. Mouse survival rates after passive transfer of 100 μg of MAb 3E10, 4C4, and 7C2 were 16%, 33%, and 8%, respectively, whereas no mice survived after injection with MAbs 7H5 or 8F1, or with the anti-HIV antibody used as a control (Figure 5, Table 5). However, increasing 7C2, 7H5, and 8F1 MAbs dosages to 500 μg per mouse enhanced the survival period (Figure 5, Table 5). Calculation of the χ^2^ for the survival rate, the delay until mortality, and the dose of antibody indicated a significant anti-viral activity with these MAbs (*p* < 0.05) (Table 5).

**Table 4:** Monoclonal antibodies characterization and testing of passive protection

**MAbs**	**Intracellular**^**a**^	**Surface associated**^**b**^	**IFA**^**c**^		**Affinity KD (nM)**^d^	**MAb binding domain**^**e**^	**% Mice survival rate**^**f**^
			**JEV Dengue 2 V**				
3E10	+	+	+	-	1.1	unknown	16
4 C4	+	+	+	+	56	NS1_1–143_	33
7 C2	+	+	+	+	17	NS1_224–352_	8
7 H5	+	+	+	+	6.4	NS1_224–352_	0
8 F1	+	+	+	+	5	NS1_224–352_	0

^a/b^MAbs were incubated with S2-NS1_1–352_ cells permeabilized or untreated (intracellular/surface associate) for fluorescence testing by flow cytometry (FACScan).

^c^ Immunofluorescence assay (IFA) for testing monoclonal antibodies reactivity against BHK cells infected with JEV, and C6/36 cells infected with Dengue 2 virus.

^d^ The affinity of MAbs to NS1 tested by surface plasmon resonance (SPR).

^e^ Reactivity of MAbs for S2- NS1_1–143_ or NS1_224–352_ fragments. Cells lysates were tested by Western blotting (WB). “Unknown” means did not react with both of the NS1 fragments or reduced and boiled NS1.

^f^ Six to twelve mice were administered with 100μg of 3E10, 4 C4, 7 C2 and 8 F1, respectively, and challenged by JEV SA14.

**Table 5:** Survival rate of C3H mice passively immunized with anti-NS1 monoclonal antibodies

**MAbs**	**MAb dose (μg)**	**No. of survival mice/ No. of passive transfer mice (survival rate)**	***p***^**a**^
3E10	100	1/6 (16.7%)	0.07
	500	1/6 (16.7%)	0.05
4 C4	100	2/6 (33.3%)	0.02
	500	2/6 (33.3%)	0.15
7 C2	100	1/12 (8.3%)	0.74
	500	1/6 (16.7%)	0.02
7 H5	100	0/6 (0%)	0.08
	500	0/6 (0%)	0.02
8 F1	100	0/12 (0%)	0.58
	500	0/6 (0%)	0.02
Anti-HIV	100	0/6 (0%)	

^a^*P* value was obtained by log rank test when comparing the survival curve of each group of mice which received anti-NS1 MAb with anti-HIV group.

## Discussion

Japanese encephalitis remains a serious public health threat in Asia, where more than two billion people are at risk. The complex JEV zoonotic cycle prevents any direct action to reduce viral transmission; therefore, human infection can only be prevented by vaccination campaigns with children [12]. Apart from the live-attenuated JEV vaccine SA14-14-2 developed for childhood application [11], no affordable JEV vaccine is recognized for mass vaccination. Several vaccine candidates inducing neutralizing antibodies are under clinical trial and they may meet the immunogenicity and protective capacity requirements after a single dose regimen [11]. However, the use of NS1 protein as an immunogen to reinforce the immune response to JEV structural proteins has been discussed, although the induction mechanisms of protective JEV immunity using a NS1 hexameric antigen form have not been investigated. *Drosophila* S2 cells have been used for JEV NS1 protein expression and purification from the cell supernatant harvested after serum-free cell culture [27]. Previous recovery of DENV E protein crystals in S2 cells indicated that this system expressed native-like and well-folded glycoproteins [29]. More than 50 mg L^–1^ of the hexameric form of NS1 glycosylated protein were expressed in the cell culture supernatant [27], which was shown in this study to contain carbohydrates similar to the native protein produced in infected cells [30] and was suitable for recombinant vaccine subunit preparation. In this study, two doses of 1 μg of glycosylated hexameric JEV NS1 were used to immunize mice. High antibody titers were detected in the sera of two groups of mice immunized with aqueous or emulsified NS1 with ISA-51-VG formulations. The adjuvant ISA-51-VG was a mix of mineral oil and a surfactant, which has been previously tested for human vaccination [31]. This adjuvant was tested for its capacity to boost the T-cell response against NS1 in immunized mice. The titers of IgG1 subclass were 4–9 times higher than those of IgG2a in both group**s** of mice, while titers of IgG1 in mice immunized with adjuvanted NS1 were 2–4 times higher when compared with those of mice immunized with aqueous NS1, whereas IgG2a titers remained low. Similarly, 100 times higher IgG1 titers compared with IgG2a were observed in mice that were immunized with *E. coli*-expressed JEV NS1 protein mixed with Freund’s adjuvant [32]. This difference was presumably due to the use of Freund’s adjuvant, the high antigen dose and the number of antigen injections (three instead of two). In vitro splenocyte stimulation by NS1 elicited T-cell proliferation and IFN-γ secretion, although higher IFN-γ secretion was observed in mice immunized with aqueous NS1. These results suggested that the NS1 proteins are engulfed by antigen presenting cells (APCs) in vivo. The peptides derived from NS1 digestion are subsequently presented by MHC class II molecules to T helper cells. The IgG1 subclass antibody response corresponds to Th2 cell activation, whereas the IgG2a response reflects Th1 cell activation. The IFN-γ produced by Th1 cells inhibits Th2 cell proliferation and IgG1 production [30]. These results indicate that NS1 immunization elicited both Th1 and Th2 cell responses and that ISA-51-VG improved IgG1 production, and reduced Th1 activation and INF-γ expression. Mice inoculated with TBE NS1, which was expressed by a recombinant adenovirus, stimulated in vivo IFN-γ production [17]*.* A previous study showed that soluble DENV-1 NS1 is a lipoprotein [4] and was internalized by mouse hepatocytes in vivo and by cultured cell lines in vitro [33]. We observed that purified soluble JEV NS1 attached to many types of cell membranes in vitro and was subsequently internalized (Li and Deubel, unpublished data). This feature may at least partially explain why aqueous NS1 rapidly stimulated the Th1 cell response via APCs, whereas NS1 mixed with ISA-51-VG was slowly released from the emulsion and preferentially activated a Th2 response [31,34]. The cell-mediated immune response induced by SA14-14-2 immunization and NS1 stimulation was also studied. Compared with the NS1 group, the SA14-14-2 group showed higher cell proliferation SI but lower IFN-γ production. These differences may be due to different epitope presentation from native protein or replicative virus cytokine profiles in response to NS1 and SA14-14-2 immunization, respectively. IL-2 was detected in SA14-14-2, but not in NS1-immunized mice (data not shown), confirming a Th1 stimulation pathway induced by replicative viruses. Production of cytotoxic T lymphocytes (CTLs) is another protective immune response induced by NS1 immunogens issued from replicative viruses [35]. Mice primed with JEV-infected cells [36] or JEV NS1 expression recombinant viruses [37] stimulated mice to generate CTLs against JEV. Further study showed that CTLs recognize peptides derived from NS1 and NS3 [35]. One study showed that NS1-expressing DNA immunogen could stimulate CTLs against JEV in mice, but induced low protection [38]. However, we did not expect any CTL response since NS1 was not presented in a replicative system that could stimulate class I antigen presentation.

The protective immune responses elicited against the purified JEV NS1 hexameric protein was investigated by challenging mice with JEV SA14 injected i.n. Immunization with recombinant subunits required several injections, but the mice were not highly susceptible to JEV at the end of the immunization schedule. However, the i.n. method adopted in this study killed more consistently mice aged over three months than i.p. inoculation (Additional file 2: Figure S2A and B). Another approach would have been to infect mice by an intracerebral or an i.p. route [39], but we felt these techniques did not simulate a natural transmission, as they mechanically break the blood brain barrier. In our study, two doses of 1 μg of hexameric NS1 emulsified in adjuvant provided significant protection (83%) against viral challenge when compared with soluble NS1 (72%), as it reduced morbidity and mortality and increased the survival period after infection. Anti-NS1 immune responses were induced by YFV, DENV, TBEV and WNV immunization with NS1 protein, an NS1-encoding DNA gene, or viral vectors expressing NS1 [13,14,18]. Mouse immunization with JEV NS1 produced in *Spodoptera frugiperda Sf*9 insect cells induced little or no protection [25], whereas NS1 carried by viral vectors induced an antibody response [26,37], but low protection [26,37]. DNA vaccination of mice with the NS1 gene [40] induced more than 80% protection, whereas DNA vaccination with the NS1-NS2A gene protected only 20% of mice against a JEV challenge [39]. Interestingly, 100 μg of non-folded and non-glycosylated JEV NS1 was produced in *E. coli* and injected three times to induce protective immunity in 87.5% of vaccinated mice [32]. Recently, a study using protein E fragments or NS1 produced in *E. coli* and injected at 50 μg seven (i.n.) or five (i.p.) times showed better protection when the proteins were injected i.n. rather than i.p. [41]. Only two much lower doses of the hexameric NS1 form provided protective effects, which may be due to the elicitation of antibodies that bind to conformational epitopes.

Higher antibody titers in the mouse group with a higher survival rate suggested that NS1 had induced an antibody response that might play an important role in preventing JEV infection. In order to verify this hypothesis, different antibody doses induced during mouse immunization were injected into naïve mice. One-month-old mice were used for the adoptive immunity test, so it was possible to apply an i.p. challenge in C3H mice, which caused 100% mortality with a low dose of virus [28]. Passive transfer of 100 μl of anti-NS1 antisera (titrated 1:3000) to one-month-old mice protected 100% from morbidity and death, whereas 30 μl was less protective and 10 μl was not protective, suggesting that an important NS1 function in JEV pathogenesis could be inhibited by IgG-NS1 immunocomplexes. However, anti-NS1 has no neutralizing activity on virus infection in vitro*.* The antibody-dependent complement-mediated cytolysis of JEV-infected cells associated with anti-NS1 antibodies was demonstrated by in vitro experiments [42]. Another study showed that the depletion of C3 complement components in mice did not affect the anti-NS1 passive protection capacity against YFV [22]. In addition, immunization with a genetic deletion in C5 using an adenovirus expressing NS1 protected mice from a TBEV lethal challenge [16]. These experiments suggested that antibody-dependent complement-mediated cytolysis may play a minor role in antibody-associated immune protection in vivo. However, WNV anti-NS1 MAb through Fc-γ receptor-dependent and C1q-independent pathways could be an alternative protection route involving the scavenging of infected cells by macrophages [23,24]. In our study, five JEV anti-NS1 MAbs generated in a previous study [27] that exhibited a high binding affinity against different NS1 epitopes were used in a passive protection test. Two MAbs exhibited no capacity to prevent death, although they significantly extended survival by 2–6 days. MAb 3E10 was directed against a conformational epitope, while MAb 4C4 recognized the NS1 N-terminus and 7C2 recognized the NS1 C-terminus and provided significant delay and prevention against JEV mortality. However, 500 μg of MAbs 7H5 and 8F1 significantly delayed death after JEV infection in a dose-dependent manner and were probably directed against a linear epitope of the C-terminus fraction of NS1 that cross-reacted with DENV NS1 (Tables 4 and 5). Whether those MAbs recognize the same epitope is not known, but they presented different NS1 binding affinities. Interestingly, anti-WNV MAbs that bound to a similar NS1 fragment also provided mice with up to 90% protective immunity from a lethal challenge [23]. Identification of the epitope(s) targeted by the three JEV anti-NS1 MAbs would facilitate further study of the NS1 antibody interaction and its function in anti-flaviviral immunity. Further study using a mixture of those MAbs and their possible role in scavenging NS1-IgG complexes would bring a better understanding of the function of anti-NS1 antibodies in protective immunity.

The mechanism by which NS1 contributes to in vivo flaviviral pathogenesis is largely unknown, which hinders the elucidation of the mechanism whereby anti-NS1 provides protective activity. Anti-NS1 antibodies interact with protein NS1′, an elongated form of NS1 found in flaviviruses of the JEV serogroup [43], which is involved in neuroinvasion by WNV subtype Kunjin [44]. However, the mechanism involved remains unknown. It is likely that, in addition to interacting with NS1, anti-NS1 antibodies may block the in vivo deleterious activity of NS1′ in WNV and JEV infections. Understanding this interaction would open up new research avenues.

NS1 alone cannot induce a sterilizing immunity, but it contributes to the consolidation of flavivirus neutralizing immunity that is primarily elicited by protein E. Several vaccine candidates are known to provide better protection when NS1 is included in the vaccine preparation [38,45-50]. A recent study demonstrated the enhancement of anti-E antibody neutralization titer by mouse co-immunization of protein E with NS1, suggesting that NS1 might be usefully added to the viral structural components in a JEV vaccine [51].

A previous study showed that autoantibodies induced by DENV NS1 recognized coagulation factors including fibrinogen and platelets, as well as integrin/adhesion proteins [8]. Binding of anti-DENV NS1 antibodies to endothelial cells induced inflammation and apoptosis [10]. The possibility that other flaviviral NS1 proteins may elicit autoantibodies when used as components of a vaccine should not be underestimated. Whether JEV NS1 induced autoantibodies needs further study. However, JEV anti-NS1 antibodies did not bind to endothelial cells like DENV anti-NS1 antibodies did [10] and JEV infection does not cause hemorrhagic manifestations, minimizing the role of NS1-elicited autoantibodies.

In summary, immunization with a hexameric NS1 elicited Th2 and low Th1 cell responses, and induced a partial protective immune response in a mouse model. The ISA-51-VG adjuvant improved the performance of NS1 immunization by contributing to the production of higher antibody titers and increased the mouse survival rate. Passive transfer of sufficient anti-NS1 antibodies provided full protection to mice from lethal JEV challenge. However, anti-NS1 MAbs provided poor protection, although they significantly extended the survival period before death. These results support the hypothesis that anti-NS1 antibodies participate in immune protection against JEV infection.

## Materials and Methods

### Cells and viruses

Baby hamster kidney (BHK-21) cell lines were cultured in Dulbecco’s modified Eagle’s medium (DMEM, Invitrogen, Carlsbad, CA, USA) containing 3% fetal bovine serum (FBS), 100 U penicillin, and 100 μg streptomycin at 37 °C in 5% CO_2_. The JEV SA14 strain was obtained from the National Institute for the Control of Pharmaceutical and Biological Products (Beijing, China). The attenuated vaccine strain JEV SA14-14-2 was obtained from the Chengdu Institute of Biological Products (Chengdu, China). BHK-21 cells were infected at a multiplicity of infection of 0.1 pfu cell^–1^. *Drosophila* S2 cells were purchased from Invitrogen (Carlsbad, CA, USA) and cultured in Schneider’s *Drosophila* medium (Invitrogen) with 10% FBS, 50 U penicillin, and 50 μg streptomycin, and incubated at 28 °C.

### NS1 expression and purification from *Drosophila* S2 cells

NS1 protein expression and purification procedures were conducted according to previously published methods [27]. Briefly, the JEV Nakayama strain genes encoding the full-length 352 amino acids of NS1 and its fragments, containing N-terminus amino acids (1–143) and C-terminus amino acids (224–352), were amplified by RT-PCR and inserted into the pMT/Bip/V5-His A plasmid vector to produce pMT-NS1_1-352_, pMT-NS1_1-143_, and pMT-NS1_224-352_, respectively. *Drosophila* S2 cells were co-transfected with plasmids pMT-NS1_1-352_, pMT-NS1_1-143_, or pMT-NS1_224-352_, and pCoblast (Invitrogen). S2 cells were selected using blasticidin. To improve NS1 production, cell populations were cloned by limit dilution to generate an S2-NS1 cell clone expressing high levels of NS1. Cells were cultured in Express Five medium (Invitrogen) without FBS in a Wave Bioreactor™ (GE Amersham, Uppsala, Sweden). Cell supernatants were collected, filtered, and concentrated, and the buffer was exchanged. The resultant fluids containing NS1 were loaded onto a chelating sepharose column (GE Amersham). The column was washed with binding buffer and the recombinant protein was eluted. After nickel ion column chromatography, the protein was applied to a size exclusion Superdex 200 10/300 column using the AKTA Purifier System (GE Amersham).

### Carbohydrate analysis of JEV NS1 secreted from *Drosophila* S2-NS1 cell clone

The NS1 denatured monomeric form was digested by Endoglycosidase H (Endo H) or Peptide: N-glycosidase F (PNGase F) (New England BioLabs, Beverley, MA, USA) according to the manufacturer’s instruction. Briefly, 3 μg of native or denatured NS1 were mixed with 6,000 units of Endo H or 7,000 units of PNGase F in reaction buffer, and incubated at 37 °C for 3 hr. After digestion, the reaction mixtures were added to equal volumes of 2 times loading buffer, heated at 95 °C for 5 min, and separated by 12.5% SDS-polyacrylamide gel electrophoresis (SDS-PAGE).

Glycosylation differentiation: The DIG glycan differentiation kit (Roche, Mannheim, Germany) was used for NS1 glycan differentiation. The NS1 proteins and control glycoproteins were transferred onto nitrocellulose membranes after gel electrophoretic separation. The membranes were incubated in blocking buffer for 1 hr, washed twice in TBS (Tris buffered saline) and once in buffer 1 (TBS; 1 mM MgCl2, 1 mM MnCl2, 1 mM CaCl2, pH 7.5) and incubated each 1 hr with digoxigenin-labeled GNA, SNA, MAA, and DSA. The membranes were washed three times with TBS, then anti-digoxigenin-alkaline phosphatase was added and incubated for 1 hr. The membranes were washed three times in distilled water and 1 ml of alkaline phosphatase substrate (Promega, San Luis Obispo, CA, USA) was added to each membrane for staining.

### Western blotting

The NS1 protein sample was boiled at 95 °C for 5 min in a reducing SDS loading buffer, then separated by 10% SDS-PAGE. The protein was transferred to nitrocellulose membranes that were blocked with 5% non-fat milk in TBS/Tween-20 before being incubated with a 1:1000 dilution of anti-V5 tag or anti-NS1 MAbs. After washing, the membranes were incubated with a 1:3000 dilution of alkaline-phosphatase-conjugated secondary antibodies (Promega). Signals were detected based on the coloration of an alkaline phosphatase dark blue substrate (Promega).

### Antibody titration

The anti-NS1 antibody titers were tested by ELISA. One hundred ng per well of purified NS1 protein was coated onto 96-well plates (Corning Costar, Lowell, MA, USA) that were incubated overnight at 4°C. Plates were washed and blocked with PBS/3% BSA. A series of 1:2 dilutions (from 1:100 to 1:204,800 in PBS/1% BSA) of mouse serum was added to the wells and incubated for 1 h at room temperature (RT). Plates were washed before adding horseradish-peroxidase-labeled goat anti-mouse IgG (H + L), IgG1, IgG2a, (Southern Biotech, Birmingham, AL, USA) that were diluted 5,000-fold. Plates were then incubated for 1 h at RT. The plates were washed again before adding ortho-phenyl diamine substrate (Sigma–Aldrich) and incubated for 10 min at RT. Enzymatic reactions were stopped with 3 M H_2_SO4 and absorbance was recorded at 492 nm using an ELISA plate reader (Molecular Devices, Sunnyvale, CA, USA)

Neutralizing antibody titers were determined in BHK cells. Pooled sera from each group were subjected to two fold dilutions in DMEM 2% FBS from 1:20 to 1:320 dilution,and mixed with 150 pfu (corresponding to 10 TCID_50_) of JEV SA14 virus in 96-well plates before incubating for 1 h at 37 °C, after which 6,000 BHK cells were added. The plates were read after 3 d, and the serum dilution that reduced 50% of the virus (TCID_50_) was recorded.

### Lymphocyte proliferation and IFN-γ secretion

Two weeks after the 2nd inoculation of NS1, PBS, or six weeks after the inoculation of SA14-14-2, DMEM with 3% FBS (complete DMEM medium), three mice were killed from each group. Mouse spleens were isolated and crushed in cell strainers (Becton, Dickinson and Company, Franklin Lakes, NY, USA) to prepare splenocyte suspensions. Erythrocytes were lysed with red blood cell lysis solution (15.5 mM NH_4_Cl, 1 mM KHCO_3_, and 0.01 mM EDTA-2Na) and centrifuged at 500 × g, for five min, the cell pellet was washed three times then re-suspended in cell culture medium. The cell concentration of each mouse was counted and diluted to 4 × 10^6^ cells ml^–1^ in RPMI 1640 medium with 10% heat-inactivated FBS to be used for lymphocyte proliferation and cytokine production assays [52]. Mouse splenocytes were cultured with 5 μg ml^–1^ of JEV NS1 protein. T-cell proliferation was quantified using the Cell Titer 96® Aqueous Non-Radioactive Cell Proliferation Assay (Promega), according to the manufacturer’s instructions. T-lymphocyte proliferation was measured using a stimulation index, which was expressed as the OD_570_ ratio of NS1-stimulated wells to that of wells without NS1 [53].

Splenocyte suspensions (0.5 ml) were dispensed into the wells of a 24-well cell culture plate. An equal volume of NS1 (10 μg ml^–1^) in medium was also added. Cells were incubated for 5 d. Cell culture supernatants were harvested and stored at −80°C before analysis. IFN-γ production was tested using a Mouse IFN-γ ELISA Kit (BD Pharmingen, San Diego, CA, USA), according to the manufacturers’ instructions [52].

### Anti-NS1 MAb preparation and characterization

Anti-JEV MAb preparation followed a previously published method [27]. Briefly, MAbs were purified from hybridoma cultured supernatants or from mouse ascitic fluid using Protein A or Protein G columns (GE Amersham). Antibodies were characterized by flow cytometry to determine cell surface and intracellular NS1 recognition. Epitope mapping was performed using an ELISA or Western blotting with full-length, and N-terminus (amino acids 1–143) and C-terminus (amino acids 224–352) NS1 fragments [27].

### Mouse immunization and challenge

The mouse experimental protocol was approved by the Institut Pasteur of Shanghai Ethics Committee for Animal Care (ID# 01–2006) and was conducted in the Institut Pasteur animal facility following the European directive 2010/63/EU on the protection of animals used for scientific purposes.

Four-week-old female C3H/HeN mice were purchased from Vital River, the Chinese representative of Charles River Laboratories (Vital River Lab Animal Technology Co., Ltd, Beijing, China). Mice were separated into four groups: a negative control group that received 200 μl of PBS i.p.; a positive control group that received one dose of 10^4^ pfu JEV SA14-14-2 vaccine i.p.; an NS1 group that was injected with 1 μg of NS1 in 200 μl of PBS (aqueous NS1); and a group injected with 1 μg of NS1 emulsified with water-in-oil adjuvant by mixing NS1 in 100 μl of PBS with an equal volume of Montanide oil (ISA-51-VG, SEPPIC SA, Puteaux, France) in a latex-free syringe. All groups were immunized by the i.p. route with two doses of antigen four weeks apart. Mouse sera were collected two weeks after the second inoculation. Four weeks after the second inoculation, mice were transferred in isolators to a biosafety level 2 animal facility, where they were challenged by i.n. inoculation of 10 μl containing 10^4^ pfu of SA14 virus in each nostril. Surviving mice sera were collected for antibody titration.

### Mouse passive protection

Four-week-old mice were treated by i.p. transfer of 100 μl, 30μl, 10μl of pooled anti-NS1 mouse sera obtained from NS1-immunized mice containing an anti-NS1 antibody titer > 1:3000, or 100 μg or 500 μg of anti-NS1 MAbs. An HIV anti-envelop IgG1 MAb (a generous gift from Pr. Paul Zhou, Institut Pasteur of Shanghai) was used as an isotype control. Passive transfer of pooled anti-JEV antisera obtained from mice vaccinated with SA14-14-2 was used as a positive control. All sera were pre-incubated at 56°C for 30 min. One hour after the sera injection, mice were challenged with an injection of 20 pfu of JEV SA14. Mouse survival rates were calculated four weeks after the challenge.

### Statistics

Cell proliferation, cytokine, and antibody titer data were analyzed by Excel and expressed as the mean ± SD. Survival analysis curves were analyzed using the log-rank test calculated by STATA version 11.1 (StataCorp, College Station, TX, USA). Figures were created using Sigmaplot (Systat Software, Inc., San Jose, CA, USA).

## Competing interests

The authors declare that they have no competing interests.

## Authors’ contributions

Conception of the study and design of the experiments: YL, VDe. Performance of the experiments: YL, CD, LP. Advice and assistance with the live-attenuated vaccine JEV: YY. Analysis of the data and statistics: YL, VDe, VDu. Writing of the paper: YL, VDe. All authors read and approved the final manuscript.

## Supplementary Material

Additional file 1 **Figure S1.** Characterization of carbohydrates of S2 cells expressing NS1. A: Purified NS1 protein was denatured and mock-treated (lane 1) or incubated with Endo H (lane 2) and PNGase F (lane 3) and the products were separated by SDS-PAGE. B: After electrophoresis, the GNA recognition control glycoprotein carboxypeptidase Y (arrow, lane 1), the DSA recognition control glycoprotein asialofetuin (lane 3), and NS1 protein (arrows, lanes 2 and 4) were transferred to a nitrocellulose membrane and incubated with digoxigenin-labeled GNA (lanes 1 and 2) or DSA (lanes 3 and 4) and further incubated with anti-digoxigenin alkaline phosphatase-labeled antibodies and with alkaline phosphatase substrate. Click here for file

Additional file 2 **Figure S2.** Susceptibility of C3H mice to JEV SA14 infection. Groups of six C3H mice infected with JEV SA14 were monitored daily for 28 days. Two groups of three-month-old C3H mice were infected with different doses of JEV SA14 by intraperitoneal (A) or intranasal (B) route. One group of one-month-old C3H mice was infected with different doses of JEV SA14 by intraperitoneal route (C). Click here for file
