# Supplementary figures and images for: Protective immunity to Japanese encephalitis virus associated with anti-NS1 antibodies in a mouse model
Source: Virol J. 2012 Jul 24;9:135. doi: 10.1186/1743-422X-9-135 (PMC3416663; doi:10.1186/1743-422X-9-135)

## Slide 1
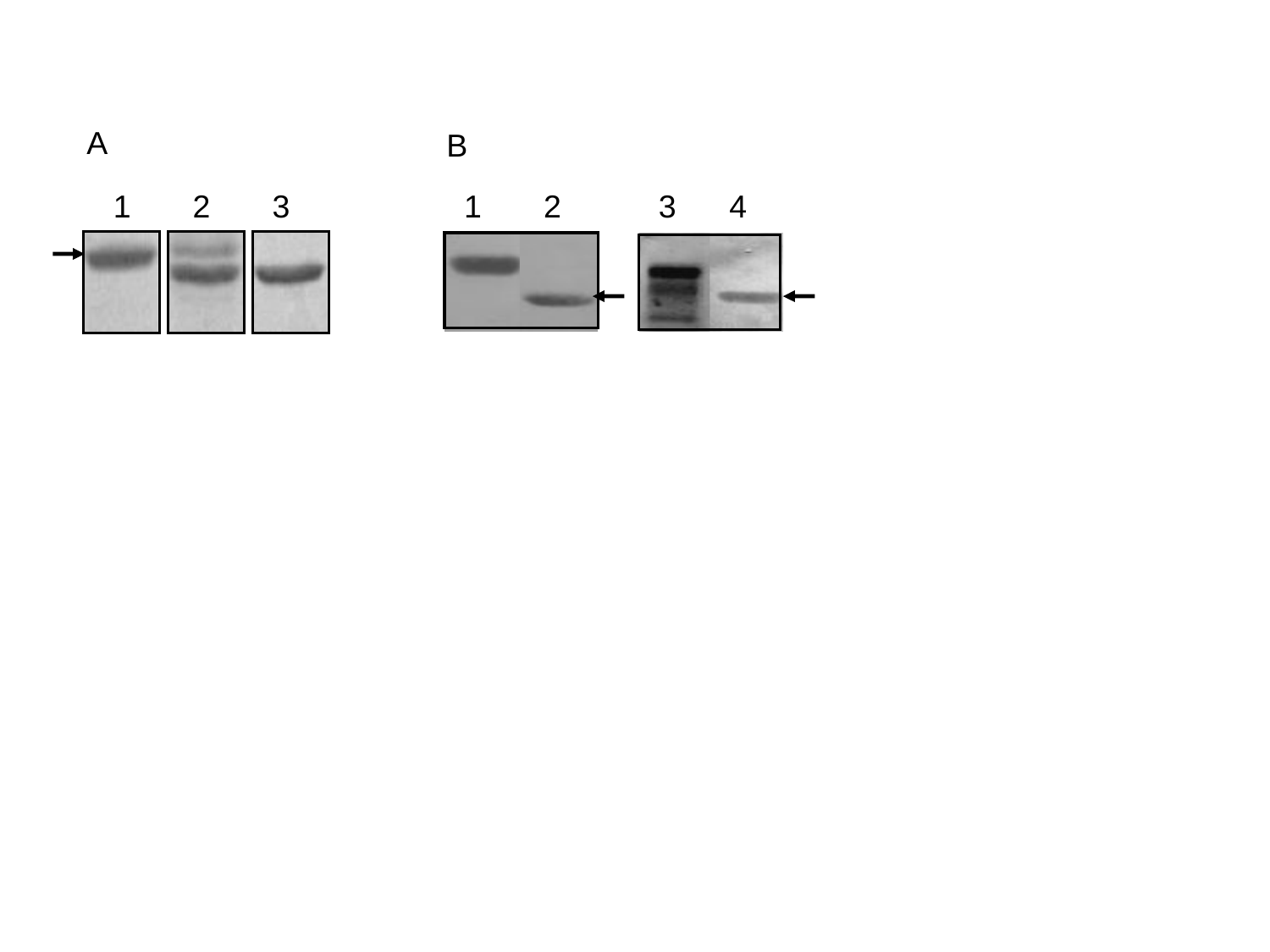

A
 1 2 3
B
 1 2 3 4

Supplement: Additional file 1 — Figure S1. Characterization of carbohydrates of S2 cells expressing NS1. A: Purified NS1 protein was denatured and mock-treated (lane 1) or incubated with Endo H (lane 2) and PNGase F (lane 3) and the products were separated by SDS-PAGE. B: After electrophoresis, the GNA recognition control glycoprotein carboxypeptidase Y (arrow, lane 1), the DSA recognition control glycoprotein asialofetuin (lane 3), and NS1 protein (arrows, lanes 2 and 4) were transferred to a nitrocellulose membrane and incubated with digoxigenin-labeled GNA (lanes 1 and 2) or DSA (lanes 3 and 4) and further incubated with anti-digoxigenin alkaline phosphatase-labeled antibodies and with alkaline phosphatase substrate. [file 1743-422X-9-135-S1.ppt]

## Slide 1
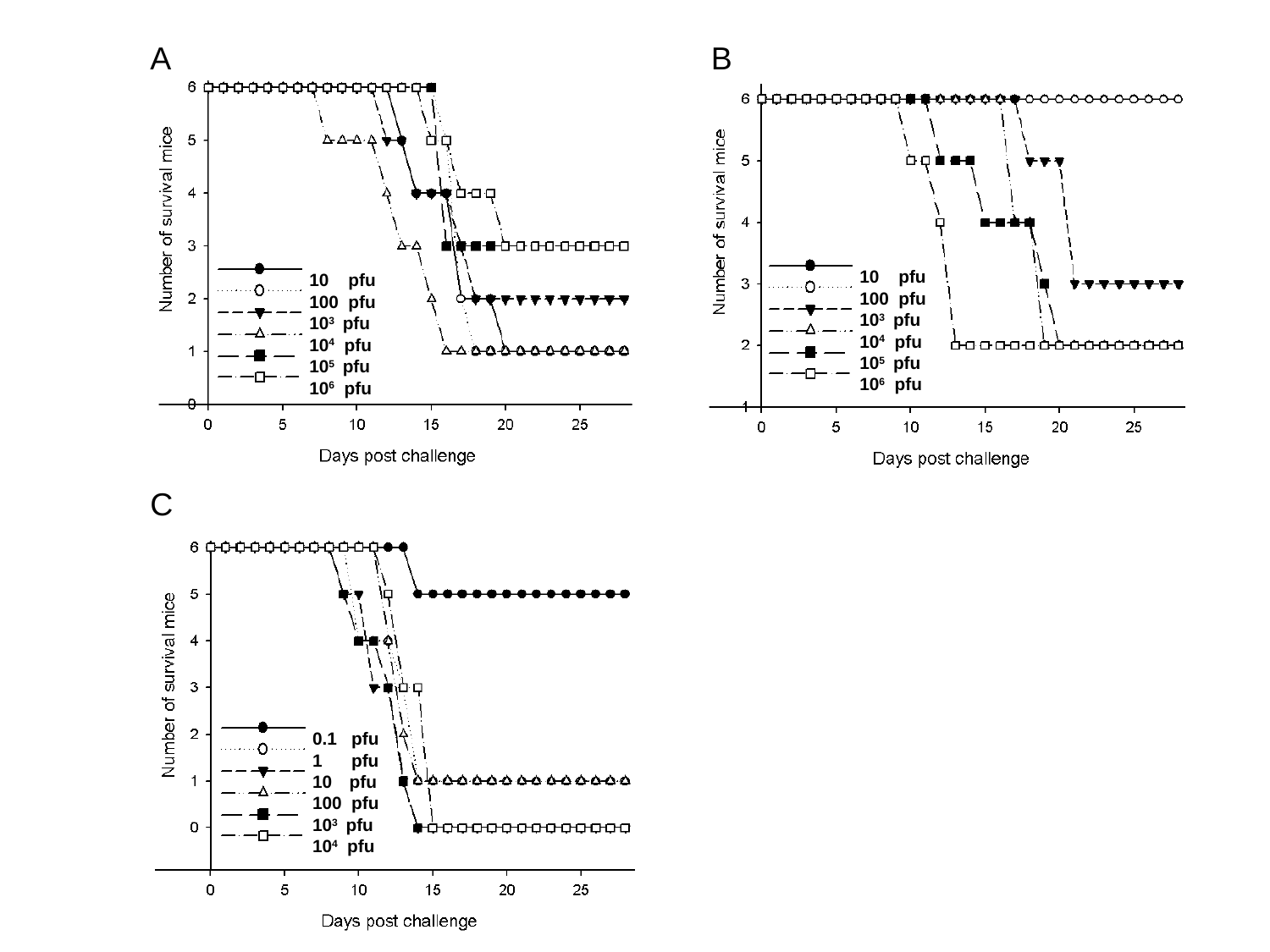

A
B
10 pfu
100 pfu
103 pfu
104 pfu
105 pfu
106 pfu
10 pfu
100 pfu
103 pfu
104 pfu
105 pfu
106 pfu
C
0.1 pfu
1 pfu
10 pfu
100 pfu
103 pfu
104 pfu

Supplement: Additional file 2 — Figure S2. Susceptibility of C3H mice to JEV SA14 infection. Groups of six C3H mice infected with JEV SA14 were monitored daily for 28 days. Two groups of three-month-old C3H mice were infected with different doses of JEV SA14 by intraperitoneal (A) or intranasal (B) route. One group of one-month-old C3H mice was infected with different doses of JEV SA14 by intraperitoneal route (C). [file 1743-422X-9-135-S2.ppt]
